# The effect of steroid hormones on the mRNA expression of oct4 and sox2 in uterine tissue of the ovariectomized mice model of menopause

**Published:** 2016-07

**Authors:** Marzieh Davoudi, Saeed Zavareh, Mohammad Taghi Ghorbanian, Seyed Hassan Paylakhi, Seyed Reza Mohebbi

**Affiliations:** 1 *School of Biology, Damghan University, Damghan, Iran.*; 2 *Institute of Biological Sciences, Damghan University, Damghan, Iran.*

**Keywords:** *Mice*, *Uterine*, *Estradiol*, *Progesterone*, *stem cells*

## Abstract

**Background:**

The uterus is a dynamic tissue responding to hormonal changes during reproductive cycles. As such, uterine stem cells have been studied in recent years. Transcription factors oct4 and sox2 are critical for effective maintenance of pluripotent cell identity.

**Objective:**

The present research evaluated the mRNA expression of oct4 and sox2 in the uterine tissues of ovariectomized mice treated with steroid hormones.

**Materials and Methods:**

In this experimental study, adult virgin female mice were ovariectomized and treated with estradiol 17β (E_2_), progesterone (P4), and a combination of E_2_ and P4 (E_2_ & P4) for 5 days. Uterine tissues were removed, and immunofluorescent (IF) staining and quantitative real-time PCR of oct4 and sox2 markers were performed.

**Results:**

IF showed oct4 and sox2 expression in the uterine endometrium and myometrium among all groups. The mRNA expression of oct4 (p=0.022) and sox2 (p=0.042) in the E_2_-treated group significantly were decreased compared to that in the control group. By contrast, the mRNA expression of oct4 and sox2 in the P4 (p=0.641 and 0.489 respectively) and E_2_ & P4-treated groups (p=0.267 and 0.264 respectively) did not show significant differences compared to the control group.

**Conclusion:**

The results indicate ovarian steroid hormones change the expression of oct4 and sox2 in the mice uterine tissues, which suggest the involvement of steroid hormonal regulation in uterine stem cells.

## Introduction

Uterine endometrium is a highly remodeled tissue undergoing cycles of growth, differentiation, and physical shedding (in humans and primates) or apoptosis (in rodents) under the influence of ovarian sex steroidal hormones, mainly, estradiol (E_2_) and progesterone (P4), during each reproductive cycle (1-4). E_2_ and P4 are responsible for the preparation of endometrium to establish a successful blastocyst implantation. After menstruation, E_2_ is essential to regenerate the endometrium and prepare it to respond to P4 after ovulation (5-7). 

P4 is essential for appropriate secretory transformation of glands and stromal decidualization to facilitate implantation (8). The effects of estrogen and progesterone are mediated through their nuclear receptors as transcriptional modulators to control different gene expression (4). The endometrium (humans and mice) contains a small population of epithelial and stromal stem cells, which are likely responsible for cyclical endometrial regeneration (9).

Octamer-binding transcription factor 4 (oct4, also known as POU5f1) belongs to the Pit-OCT-Unc transcription factor family, which is the main self-renewal regulator and pluripotency of embryonic stem cells (ESCs) (10-14). The expression of oct4 is promptly inhibited at the beginning of cell differentiation (11, 12). Sox2, a member of the *sox* HMG box family of transcription factors (SRY-related HMG-box gene 2), is required in early embryonic development to maintain pluripotency and self-renewal of ESCs (15). Sox2 regulates both embryonic and adult stem cell differentiation and proliferation (16, 17). Oct4 forms a complex with sox2 and functions to maintain the self-renewal and pluripotency of ESCs by either activating or suppressing the target gene expression (18).

Uterine stem cells respond to changes in circulating steroid hormones and affect the population of progenitor cells, which in turn become committed to particular types of differentiated cells, such as epithelial, stromal, and vascular cells, within a specific microenvironment, subsequently allowing a rapid reconstruction of the endometrium tissue to support pregnancy (19). it was suggested that ovarian derived steroid hormones are likely to stimulate the proliferation of endometrial stem cells or their neighboring niche cells (20). 

However, it is not known how estrogen and progesterone regulate uterine stem cell activity. Therefore, the present study aimed to determine whether genes expression of oct4 and sox2 at mRNA and protein levels in mouse uterine tissue affected by exogenous steroid hormones.

## Materials and methods

**Reagents**

All reagents were purchased from Merck Germany unless otherwise stated.

**Animal and experimental design**

In this experimental animal study, adult virgin female mice from the NMRI (National Medical Research Institute) (8-10 wks old; n=12), were purchased from Pasteur Institute, Iran. The mice were cared for and used in accordance with the Declaration of Helsinki of Laboratory Animals ethic, and has been approved by the Animal Care and Use Committee of Damghan University (No:211/2016). They were housed in standard conditions at 24±2^o^C with a 12 hr dark/light cycle and fed food and water *ad libitum* for at least one month in the central mice room. 

The animals were weighed before and after the experiment. Estrus cycles of mice were determined by evaluating their vaginal smear cytology, and those on diestrus phase were selected (21, 22). Hormones were dissolved in ethanol, then diluted in mineral oil to reach ﬁnal concentrations (4, 23). Bilateral ovariectomy was performed using previously described methods (24). The mice were randomly distributed into four groups: control group, ovariectomized mice without treatment; E_2_ group, ovariectomized mice treated with E_2_ (8 µg/kg/BW/IP; Sigma-Aldrich, St. Louis, USA) for 5 days consecutively; P4 group, ovariectomized mice treated with P4 (50 µg/g/BW/IP; Sigma-Aldrich, St. Louis, USA) for 5 days; and E_2_ & P4 group, ovariectomized mice treated with a combination of E_2_ and P4, in which E_2_ (8 µg/kg/BW/IP) was received on the first day and P4 (50 µg/g/BW/IP) from the second until the fifth day. 

**Tissue preparation**

Sampling was performed 1 day after final injection. The mice were sacrificed by cervical dislocation and middle portions of uterine horns were harvested immediately and cleansed of fat then processed for further analysis. Each experiment was repeated at least thrice.

**RNA extraction, reverse transcription, and quantitative real-time PCR**

Total RNA of uterine tissues was extracted using RNeasy Plus Mini Kit (Qiagen, Valencia, CA, USA) in accordance with the manufacturer’s instructions. For quantitating RNA, spectrophotometrically reading was taken at wavelengths of 260 nm and 280 nm. Also, RNA quality was assessed using a density ratio of 28 S to 18 S rRNA bands. First-strand complementary DNA (cDNA) from 1 μg of total RNA was synthesized using random hexamer primer (Fermentas-S0142, USA), RevertAid M-Mul V Reverse Transcriptase (Fermentas- EP0441, USA), Ribolock RNase Inhibitor, dNTP Mix (Fermentas, R0191, USA), 5X Reaction Buffer for RT (Fermentas, USA), and RNase-free water in accordance with the manufacturer’s instructions for RT-PCR (Fermentas, USA). Real-time PCR was performed on a Rotor-Gene 6000 machine (Curbet 3000, Qiagene) by using EvaGreen qPCR Mix Plus (Solis Biodyne) with the primers (Generay Biotech) listed in table I. 

The resultant mean threshold cycles were used for further analysis. The thermal profile was 94^o^C for 10 min, 40 cycles of denaturation at 94^o^C for 15 sec, annealing at 58^o^C for 25 sec, and elongation at 72^o^C for 25 sec. Cycle of threshold (Ct) values were calculated using Rotor-Gene software. Oct4 and sox2 expression data were normalized to ACTIN-B as internal control, and gene expression was analyzed by REST software (Corbett Life Science, Qiagen Company, USA).

**Immunofluorescent localization**

Fresh tissue samples were fixed in paraformaldehyde for 24 hr at room temperature. Subsequently, tissue samples were dehydrated in ascending-graded alcohols and xylene then embedded in paraffin. Afterward, 5 μm cross sections of uterine tissues were prepared and mounted on slides then deparaffinized in xylene and rehydrated in descending-graded ethanol. These sections were washed with distilled water for 6 min for Immunofluorescent (IF) analysis of oct4 and sox2. The sections were boiled in deionized water supplemented with 0.01 molar sodium citrate antigen retrieval solution (pH=6) by using a microwave oven at 720, 360, and 180 W at 3 min intervals. 

The slides were allowed to gradually cool at room temperature for 20 min and washed with TBS and treated with 10% normal goat serum in PBS for 1 hr; the samples were then incubated overnight at 4^o^C with primary antibodies for oct4 (Anti-oct4 antibody, Abcam, ab18976, UK) or sox2 (Anti-sox2 antibody, Abcam, ab97959, UK) diluted in PBS in accordance with the manufacturer’s instructions. For negative controls, the primary antibodies were omitted. The sections were then washed thrice with PBS and incubated with Goat Anti-Rabbit IgG Fluorescein conjugated secondary antibody (FITC, AP132F, CHEMOCON) for 1 hr at 37^o^C; afterward, these sections were washed again twice with PBS for 5 min and then evaluated under a fluorescence microscope (Nikon Eclipse, E600, Japan). 

**Statistical analysis**

All data were presented as mean±SD. The real-time PCR results were analyzed by REST software. Statistical analysis of body weights of experimental groups was performed using SPSS-ver.16 software package for windows (SPSS Inc, Chicago, IL, USA). For each analyzed variable, data were submitted to normality analysis (*Kolmogorov-Smirnov* test) and One-way analysis of variance (ANOVA) and Tukey's HSD Post Hoc test was used to compare between the groups. A significance level of (p<0.05) was considered to indicate as statistical difference.

## Results

**Figure 1:**
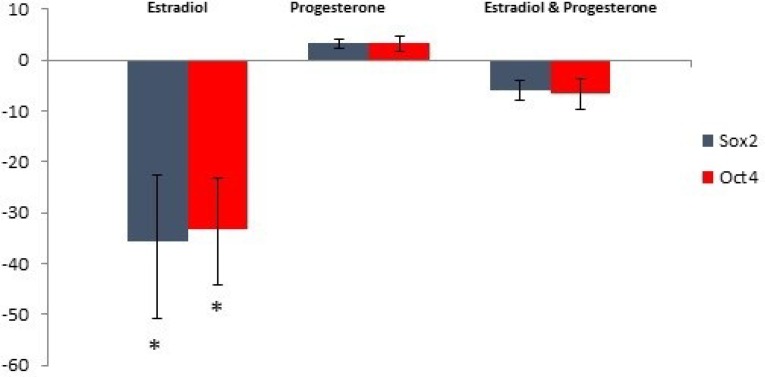
mRNA expression levels of oct4 and sox2 in uterine tissues treated with E_2_, P4, E_2_ & P4 compared to those in the control group. Data are expressed as mean±SD.* indicates significant difference (p<0.05).

No significant difference was existed in the weight of experimental mice groups before (F(4,40)=0.911, p=0.467) and after ovariectomy (F(4,40)=1.39, p=0.252). There was not a statistically significant difference between groups before (F(4,40)=2.07, p=0.42) and after (F(4,40)=1.01, p=0.37). The results showed that extracted RNA had OD260/OD280 and OD260/OD230 values of 1.8-2.0 and 1.7-2.4 respectively. Quantitative real-time PCR revealed the mRNA expression of oct4 and sox2 in all the groups (Figure 1). Comparison of the normalized Ct values demonstrated that the mRNA expression of oct4 (p=0.022) and sox2 (p=0.042) in the E_2_ group significantly decreased compared to that in the control group.

**Figure 2:**
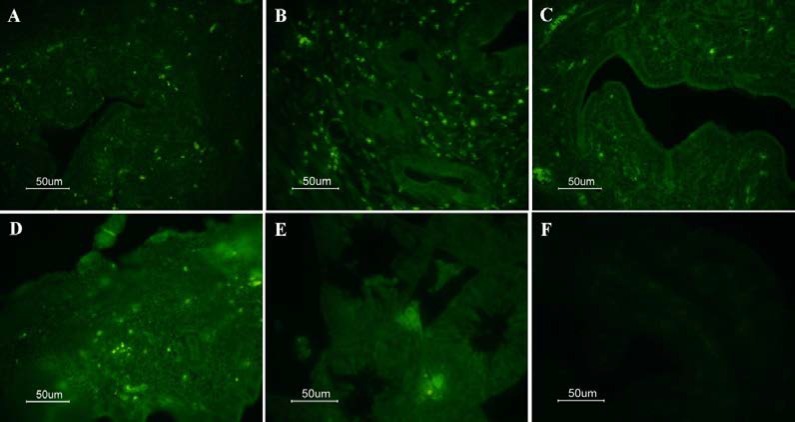
Oct4 immunoreactive cells of uterine tissue from ovariectomized mouse

**Figure 3:**
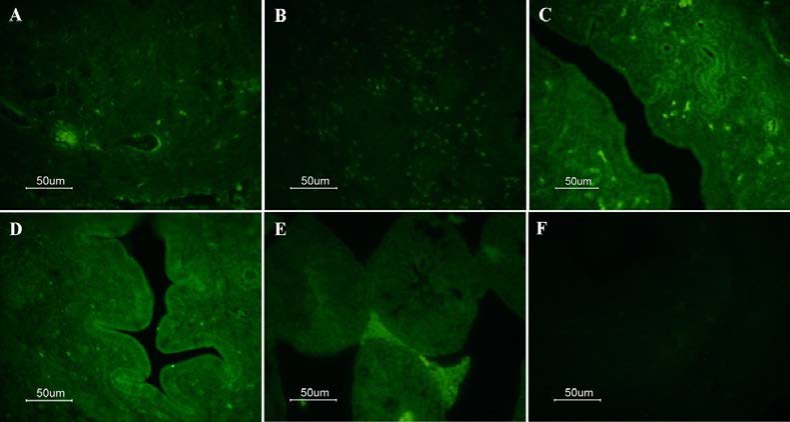
Sox2-immunoreactive cells of uterine tissue from ovariectomized mouse

By contrast, no significant difference was existed in the mRNA expression levels of oct4 (p=0.641) and sox2 (p=0.489) between the P4 and control groups. Furthermore, the mRNA expression levels of oct4 (p=0.267) and sox2 (p=0.264) in the E_2_ & P4 group did not show any significant difference with the control group. IF results indicated the expression of pluripotency markers oct4 and sox2 in the endometrium and myometrium of the uterine tissues among all the experimental groups (Figures 2, 3).

**Table I:** List of primers

**Primer**	**Sequence**	**Primer size**	**Product size**	**TM**	**Accessions Numbers**
Oct4-F	5- CGGAAGAGAAAGCGAACTAGC-3	21	107	59.23	NM_001252452.1
Oct4-R	5- ATTGGCGATGTGAGTGATCTG-3	21		59.53	
Sox2-F	5- CAGCATGTCCTACTCGCAGC-3	20	115	60.55	NM_011443.3
Sox2-R	5- GGAGTGGGAGGAAGAGGTAAC-3	21		57.59	
Actin-β-F	5- GATTACTGCTCTGGCTCCTAG-3	21	147	54.51	NM_007393.3
Actin-β-R	5- GACTCATCGTACTCCTGCTTG-3	21		56.02	

**Table II:** Body weights of experimental groups

**Group**	**BW (g)**	**AW (g)**	**PW (g)**	**SW (g)**
Control	28.55 ± 1.62	28.01 ± 2.092	33.57 ± 1.83	33.57 ± 1.83
E_2_	29.61 ± 2.16	28.98 ± 2.12	33.94 ± 2.17	33.56 ± 2.04
P4	28.06 ± 2.06	27.19 ± 1.97	32.19 ± 2.06	32.07 ± 2.14
E_2_ & P4	28.45 ± 3.71	27.92 ± 3.56	33.13 ± 2.99	33.98 ± 2.86

Data are expressed as mean±SD.

E_2_: 17β-estradiol

P4: progesterone

BW: body weight before ovariectomy

AW: body weight after ovariectomy

PW: pre-treatment body weight

SW: post-treatment body weight (before sacrifice)

A; control

B; E_2_ group

C; P4 group

D; E_2_ & P4 group

E; adult testis tissue as positive control

F. Uterine tissue with primary antibody was omitted as negative control.

A; control

B; E_2_ group

C; P4 group

D; E_2_ & P4 group

E; adult testis tissue as positive control

F. Uterine tissue with primary antibody was omitted as negative control.

## Discussion

The present study investigated the effects of exogenous E_2_ and P4 alone or in combination on the expression of pluripotency stem cell markers (sox2 and oct4) in the uterine tissues of mice menopause model for the first time. The results of the present study showed that, the genes of oct4 and sox2 expressed at mRNA and protein levels in the uterine endometrium and myometrium of all treated groups. Our results were in agreement with other investigations that indicated the expression of pluripotent stem cells in human and mice uterine tissue during normal menstrual and estrous cycles respectively (25, 26). Also, the results indicated that the mRNA expression of oct4 and sox2 significantly decreased in E_2_ treated group compared with the control group, whereas no significant differences were existed in P4 and E_2_ & P4 treated groups compared to the control group. In this sense, it was showed that exogenous steroid hormones can affect the uterine morphology and histology of ovariectomized mice ((27). 

Estrogens act via two kinds of cellular pathways, namely, estrogen receptor (ER)-dependent and ER-independent pathways. Low estrogen concentrations up to 20 nM act via ER-dependent pathways by binding to steroid receptors (ERs) of nucleus to form ER-estrogen complexes. By contrast, high estrogen concentrations act through ER-independent pathways (28). Moreover, ER-estrogen complexes may increase the oct4 expression directly by binding to the *oct4* gene promoter region and activating gene transcription or indirectly by affecting the histone stability of *oct4* gene promoter via histone acetylation and inducing *oct4* gene promoter activation (28). 

In ER-independent pathways (with high estrogen concentrations), estrogens may be metabolized into some metabolites, such as reactive oxygen species, which may induce the activation of nuclear factor kappa-light-chain-enhancer of activated B cells (NF-κB). NF-κB activates histone deacetylase and inhibits *oct4* gene transcription (29-31). The present study showed high dosage of E_2_ reduces the gene expression of oct4 and sox2. it was demonstrated that the mRNA expression of sox4 in intact mice uterine tissues was modulated at the estrus cycle by ovarian steroid hormones. So that, E_2_ reduces the mRNA expression of sox4, whereas E_2_ & P4 partially abolish the E_2_ effects (4). On the other hand, E_2_ could be responsible for uterine endometrial reconstruction through stimulating proliferation and differentiation of stem cells, so that under cyclic increase of the serum level of E_2_, uterine stem cells migrate and become progenitor cells which were committed to generate specific types of differentiated cells (19, 32). Thus, it is possible that E_2_ induces differentiation of pluripotency stem cells to differentiated cells which in turn lead to reduce the expression of mRNA of oct4 and sox2.

In normal cycling mice, serum P4 plays a critical role to induce the mRNA expression of sox4 (4). By contrast, exposure to E_2_ before treatment with P4 resulted in the induction of progesterone receptor (PR) expression, which consequently optimized the effects of P4 on the uterine tissues (4, 33). Hunt and Clarke showed that P4 alone cannot induce the mRNA expression of sox4 in ovariectomized mice (4). This finding supports the results of present research, which showed no significant difference in the mRNA expression of oct4 and sox2 between the P4 treated group and control group. 

A slight change in the mRNA expression pattern of oct4 and sox2 in the E_2_ & P4 treated group was noted compared to the control group. Such difference depends on the duration of combination therapy, dosage of hormones, and dominant hormone type. In the E_2_&P4 treated group, E_2_ was used for a short period of time, which may reduce the PR expression in uterine tissues and influence the effect of P4 on the uterine cells; additionally, P4 through secreted proteins of stromal cells inhibited the effect of estrogen (28, 34-36). 

## Conclusion

In conclusion, the results of this study indicate that the mRNA expression of oct4 and sox2 in the uterine tissues was affected by ovarian steroid hormones. The combination therapy partially eliminated the effects of E_2_ on the mRNA expression of oct4 and sox2.

## Note

***This article extracted*** ***from M.Sc. thesis. (Marzieh Davoudi)***
